# Amino Acids at Positions 156 and 332 in the E Protein of the West Nile Virus Subtype Kunjin Virus Classical Strain OR393 Are Involved in Plaque Size, Growth, and Pathogenicity in Mice

**DOI:** 10.3390/v16081237

**Published:** 2024-08-01

**Authors:** Shigeru Tajima, Hideki Ebihara, Chang-Kweng Lim

**Affiliations:** Department of Virology I, National Institute of Infectious Diseases, 1-23-1 Toyama, Shinjuku, Tokyo 162-8640, Japan

**Keywords:** West Nile virus, Kunjin virus, glycosylation, E protein, pathogenicity, reverse genetics

## Abstract

The West Nile virus (WNV) subtype Kunjin virus (WNV_KUN_) is endemic to Australia. Here, we characterized the classical WNV_KUN_ strain, OR393. The original OR393 strain contained two types of viruses: small plaque-forming virus (SP) and large plaque-forming virus (LP). The amino acid residues at positions 156 and 332 in the E protein (E^156^ and E^332^) of SP were Ser and Lys (E^156S/332K^), respectively, whereas those in LP were Phe and Thr (E^156F/332T^). SP grew slightly faster than LP in vitro. The E protein of SP was N-glycosylated, whereas that of LP was not. Analysis using two recombinant single-mutant LP viruses, rKUNV-LP-E^F156S^ and rKUNV-LP-E^T332K^, indicated that E^156S^ enlarged plaques formed by LP, but E^332K^ potently reduced them, regardless of the amino acid at E^156^. rKUNV-LP-E^F156S^ showed significantly higher neuroinvasive ability than LP, SP, and rKUNV-LP-E^T332K^. Our results indicate that the low-pathogenic classical WNV_KUN_ can easily change its pathogenicity through only a few amino acid substitutions in the E protein. It was also found that Phe at E^156^ of the rKUNV-LP-E^T332K^ was easily changed to Ser during replication in vitro and in vivo, suggesting that E^156S^ is advantageous for the propagation of WNV_KUN_ in mammalian cells.

## 1. Introduction

The West Nile Virus (WNV) is the etiological agent of West Nile fever/West Nile encephalitis. Most (~75%) human WNV infections are asymptomatic, and 1 in 150–250 symptomatic cases develops neuroinvasive disorders [1]. Approximately 10% of patients with neuroinvasive diseases die; however, the fatality rate is age-dependent and higher in patients over 70 years of age [1]. WNV is a mosquito-borne flavivirus and a member of the Japanese encephalitis virus serocomplex, which includes other clinically important human pathogenic viruses, such as the Japanese encephalitis virus, St. Louis encephalitis virus, Usutu virus, and Murry Valley encephalitis virus [1]. The WNV was first isolated from a febrile patient in Uganda in 1937 [2]. After the 1950s, several small outbreaks of WNV infection occurred in Africa, the Middle East, parts of Europe, and India, and the virus was considered to induce a mild febrile illness (West Nile fever) [3]. However, since the 1990s, the number of severe and fatal neurological cases of WNV infection (West Nile encephalitis) has gradually increased. In 1999, a WNV circulating in the Middle East and Northern Africa was introduced into the New Continent and spread rapidly throughout the region [4,5,6].

WNV is transmitted in enzootic cycles involving *Culex* mosquito vectors and virus reservoir birds, and humans and domestic animals, such as horses, are considered incidental hosts. Humans are infected with WNV by being bitten, mainly by *Culex* mosquitoes. No specific drugs or vaccines are available for WNV infection in humans. Although several vaccine candidates against WNV are currently being developed, they have not been approved for human use [7]. WNV can be classified into nine lineages (L1-L9) [8]. L1 is the most widely distributed lineage of WNV [5]. L1 strains have been identified in many regions, including the Americas, Africa, Europe, Russia, India, the Middle East, and Australia [9]. L1 strains show highly virulent phenotypes and are involved in serious outbreaks in humans. L1 can be subdivided into three sub-lineages (L1a, L1b, and L1c). The WNV NY99 strain, a representative WNV strain isolated during the first WNV outbreak in the USA, with a highly virulent phenotype in mice, belongs to L1a. L1b is composed of a WNV subtype Kunjin virus strain (WNV_KUN_), which is unique to Australia and the only WNV lineage present in Australia [10]. WNV_KUN_ has also been isolated from Malaysia [11]. WNV_KUN_ causes only mild clinical symptoms in humans and horses, and there have been no reports of death among confirmed cases of infection [12]. These findings suggest that WNV_KUN_ may be useful for the development of a live-attenuated vaccine against WNV infection [13,14,15]. However, an outbreak of encephalitis caused by WNV_KUN_ occurred in horses in Southeastern Australia in 2011, indicating that a virulent WNV_KUN_ had emerged in the area since the early 2010s [16,17]. Moreover, Prow et al. suggested that not only less virulent but also highly virulent strains of WNV_KUN_ have circulated in Australia since the 1980s [18], suggesting that the classical WNV_KUN_ strains are not always suitable for the development of live-attenuated WNV vaccines, and comprehensive virulence analysis is also required for the development of vaccines.

The classic WNV_KUN_ strain OR393 was isolated from *Culex* mosquitoes in Australia in 1974 [19,20]. Several reports have demonstrated that the glycosylation of the potential N-glycosylation site (residues 154–156, Asn-Tyr-Ser) in the WNV E protein is partially involved in its infectivity and pathogenicity, though the modification is not required for WNV pathogenicity in birds [8,9,18,21,22,23,24,25,26]. Most WNV strains are glycosylated at position 154 of E, whereas some classical WNV_KUN_ strains are not. Previous sequence analysis of OR393 revealed that the amino acid at position 156 of the E protein (E^156^) is Phe (Asn-Tyr-Phe), indicating that the E protein of OR393 is not glycosylated as well as less virulent than classic WNV_KUN_ strains [18,19]. In this study, we focused on the OR393 strain and examined its in vitro and in vivo properties to assess its utility in the development of a live-attenuated WNV vaccine.

## 2. Materials and Methods

### 2.1. Viruses

The WNV_KUN_ OR393 strain was isolated from *Culex* mosquitoes in East Kimberley, Western Australia, in 1974 (GenBank accession No. AF196503) [19]. Large plaque-forming virus (LP) and small plaque-forming virus (SP) clones of OR393 were obtained using the limiting-dilution method as described previously [27]. Complete nucleotide sequences of the LP-F and SP-B clones were determined. A working virus stock was prepared via amplification in Vero cells.

### 2.2. Cell Culture

African green monkey kidney Vero cells (strain 9013), human neuroblastoma IMR-32 cells, and mouse neuroblastoma Neuro-2a cells were cultured at 37 °C in 5% CO_2_ in Eagle’s minimal essential medium (MEM) (Sigma-Aldrich, St. Louis, MO, USA) supplemented with 10% heat-inactivated fetal bovine serum (FBS) (CORNING, Corning, NY, USA) and 100 U/mL of penicillin–streptomycin (Nacalai Tesque, Kyoto, Japan). Mosquito Aedes albopictus-derived C6/36 cells were maintained at 28 °C under 5% CO_2_ in MEM supplemented with 10% heat-inactivated FBS and 100 U/mL of penicillin–streptomycin.

### 2.3. Plaque Formation Assay for Titration of Infectious Viruses and Analysis of Growth Kinetics

Infectious viral titers for each sample were determined using plaque formation assays. Vero cells (approximately 5 × 10^5^/well) were seeded into 12-well culture plates and inoculated with each virus for 1 h at 37 °C. Next, MEM-based overlay medium containing 1% methylcellulose (FUJIFILM Wako Pure Chemical, Osaka, Japan) and 2% FBS was added to the wells, and the cells were incubated for 5 or 6 days at 36–37 °C, after which they were fixed using a 10% formalin–PBS solution and stained with methylene blue. The diameters (width of the core of the comet-shaped plaques) of 10 plaques were measured, and the mean plaque size (mm ± SD) was calculated. Differences in mean plaque sizes were analyzed using Student’s *t*-test. The ability of WNV_KUN_ strains to grow in vitro was analyzed as previously described [28]. Briefly, cells were cultured in six-well culture plates and infected with each WNV_KUN_ strain in 3 mL of MEM supplemented with 2% FBS (2F/MEM) at a multiplicity of infection (MOI) of 0.01–0.05 plaque-forming units (PFU)/cell. Small aliquots (200 μL) of the media were collected at one-day intervals, and infectious viral titers were determined using a plaque formation assay in Vero cells, as described above. Infectious virus titers in samples from virus-inoculated mice were statistically compared using GraphPad Prism version 7 (GraphPad Software, Boston, MA, USA) and the Mann–Whitney U test. Statistical significance was set at *p* < 0.05.

### 2.4. Immunoblotting

Culture supernatants and cells were collected 24 and 48 h after virus inoculation, and the cells were lysed in RIPA Buffer (Nacalai Tesque). The supernatant and lysate samples were subjected to SDS-PAGE on a 4–12% gradient polyacrylamide gel (Thermo Fisher Scientific, Waltham, MA, USA). Immunoblotting was performed using an anti-WNV E rabbit polyclonal antibody (GTX132052; GeneTex, Irvine, CA, USA). To examine the glycosylation status of E protein, aliquots of the supernatants and cell lysates were treated with endoglycosidase H (Endo H) and peptide N-glycosidase F (PNGase F) for 90 min at 37 °C according to the manufacturer’s instructions (New England Biolabs, Ipswich, MA, USA) before Western blotting.

### 2.5. Establishment of a Reverse-Genetics System for the WNV_KUN_

A reverse-genetics system for the WNV_KUN_ OR393 large-plaque strain (LP-F; GenBank accession no. LC802099) was established as previously described [29], with some modifications (Supplementary Figure S1). Four viral cDNA fragments (A region: 1-3072, B region: 2832-6013, C region: 5721-8913, and D region: 8595-11020) were synthesized and amplified using a PrimeScript II High Fidelity One-Step RT-PCR kit (Takara Bio, Shiga, Japan). Primers used for amplification are listed in Supplementary Table S1. Each of the four PCR products was inserted into the SmaI site of the plasmid pMW119 (Nippon Gene, Tokyo, Japan) using an In-Fusion HD cloning kit (Takara Bio) and then amplified in *E. coli* STBL2 (Thermo Fisher Scientific, Waltham, MA, USA). The nucleotide sequences of the plasmid clones A^KUNV^/pMW, B^KUNV^/pMW, C^KUNV^/pMW, and D^KUNV^/pMW were verified prior to the next amplification step. The four fragments were amplified from the plasmid clones via PCR using the Q5 hot-start PCR master mix (New England Biolabs, Ipswich, MA, USA) and then concatenated to form a full-length amplicon via joint PCR using a 5′-terminal primer with a T7 promoter sequence (T7-KUNV_001f) and a 3-terminal primer (KUNV_11020r). The full-length WNV_KUN_ cDNA amplicon was transcribed using mMESSAGEmMACHINE T7 RNA transcription kit (Thermo Fisher Scientific), and after DNase I treatment and RNA purification, the synthesized RNA was transfected into Vero cells using the TransIT-mRNA Transfection kit (Mirus Bio, Madison, WI, USA), and cells were incubated for 6 days. The culture supernatant fluid was recovered, and a small aliquot was inoculated into Vero cells to amplify the recombinant WNV_KUN_ virus rKUNV. The nucleotide sequence of the recombinant virus was determined using Sanger sequencing, and no unintentional nucleotide mutations were detected.

### 2.6. Production of Point Mutant WNV_KUN_

To produce the point mutant viruses rKUNV-LP-E^F156S^ and rKUNV-LP-E^T332K^, the A-region clone A^KUNV^/pMW was amplified via inverse PCR using primers with point mutations U1433C (E^F156S^) and C1961A (E^T332K^), respectively (Supplementary Table S1). The PCR products were self-ligated and amplified in *E. coli*. The resultant clones A^KUNV_U1433C^/pMW and A^KUNV_C1961A^/pMW were used to produce recombinant WNV_KUN_ mutants, as described above. The nucleotide sequences of the mutant viruses were determined, and no unintentional mutations were detected.

### 2.7. Mouse Challenge Experiment and Sample Collection

Female ddY mice (Japan SLC, Shizuoka, Japan) were used for challenge tests. For neuroinvasive analysis, groups of mice (3 weeks old, *n* = 6) were inoculated intraperitoneally (i.p.) with 100 μL (5 × 10^4^ PFU and 5 × 10^5^ PFU) of the virus solution diluted in 0.9% NaCl solution. The mice were observed, and their body weights were measured daily for 20 days after inoculation to assess survival rates. Survival curves were compared using GraphPad Prism version 7 and log-rank (Mantel–Cox) tests. Statistical significance was set at *p* < 0.05. To analyze neurovirulence, groups of mice (4 weeks old, *n* = 6) were inoculated intracerebrally (i.c.) with 30 μL (3 × 10^2^ PFU and 3 × 10^3^ PFU) of the virus solution, and the mice were observed to determine survival rates, as described above.

For growth analysis, groups of mice (*n* = 5) were inoculated i.p. with 100 μL (1 × 10^5^ PFU) of virus solution. The serum, brain, and spleen were collected from mice at 2 and 5 days post-infection, and the infectious titer and RNA levels of the infectious virus in the samples were measured, as described above and below. Tissue weights were determined, and the tissues were homogenized in 500 μL of 2F/MEM for 30 s at 6000 rpm using Precellys Evolution Touch (Bertin Technologies, Montigny-le-Bretonneux, France). The homogenate was used to measure infectious virus titers and viral genomic copy numbers as described above and below. The nucleotide sequences at positions E^156^ and E^332^ were determined using Sanger sequencing of several brain samples.

### 2.8. Measurement of Viral Genome Copy Number

Total RNA was extracted from the serum samples using a High Pure Viral RNA Purification Kit (Roche Diagnostics, Indianapolis, IN, USA). To measure the total copy number of the viral genome in the cells and supernatant, we used the real-time RT-PCR (TaqMan) method with the probe WNV_3538p and primers WNVcom.3451f and WNVcom.3590r, as described in Supplemental Table S1. Partial cDNA of the WNV_KUN_ pAKUN clone (AY274505, nt 3301-3800) [30] was synthesized in vitro and inserted into the T7 promoter site downstream of the cloning plasmid pTAC-2 (Eurofins Genomics, Tokyo, Japan). Positive control RNA was synthesized from the plasmid using the mMASSAGE mMACHINE T7 kit, as described above. Genome copy numbers were statistically compared using GraphPad Prism version 7. Statistical significance was set at *p* < 0.05.

## 3. Results

### 3.1. WNV_KUN_ OR393 Contained Small-Sized Plaque and Large-Sized Plaque Viruses

**Figure 1:**
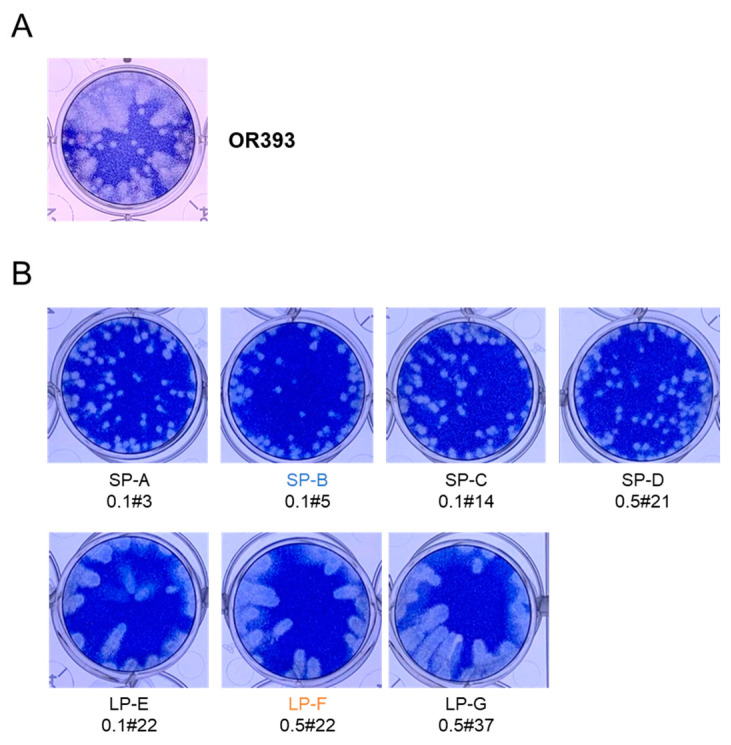
Plaque phenotypes of the original virus, WNV_KUN_ OR393 (**A**), and subcloned small-sized plaque-forming (SP) and large-sized plaque-forming (LP) OR393 viruses (**B**). Six days post-inoculation, the cells were fixed and stained.

A plaque assay was conducted using Vero cells to determine the infectious titer of the WNV_KUN_ OR393 strain (Figure 1A). The original virus solution contained at least two distinct types of viruses: small plaque-forming virus (SP) and large plaque-forming virus (LP). Single-clone viruses were obtained from the original virus solution using the limiting dilution method to determine the nucleotide sequences of the SP and LP variants. Four SP and three LP clones were obtained (Figure 1B). The complete nucleotide sequences of the two clones from each group (SP-A, SP-B, LP-E, and LP-F) were determined (Table 1). There were six nucleotide variations among the clones, but two (nucleotides 1433 and 1961) of the six sites were different between the SP and LP clones; nucleotides 1433 and 1961 were C and A, respectively, in the SP clones, and U and C, respectively, in the LP clones. The two sites were in the E protein-coding region, and amino acid residues at nucleotides 1433 (amino acid 156 in E, E^156^) and 1961 (amino acid 332 in E, E^332^) were Ser (E^S156^) and Lys (E^K332^), respectively, in the SP clones, but Phe (E^F156^) and Thr (E^T332^), respectively, in the LP clones. The other two SP and one LP clones also maintained SP-specific (C1433 and A1961) and LP-specific (U1433 and C1961) sequences at these two positions, respectively (Table 1). These results raise the possibility that these two sites may be associated with the differences in plaque morphology between the SP and LP groups.

### 3.2. Growth Ability of Small- and Large-Sized Plaque WNV_KUN_ Clones In Vitro

**Figure 2:**
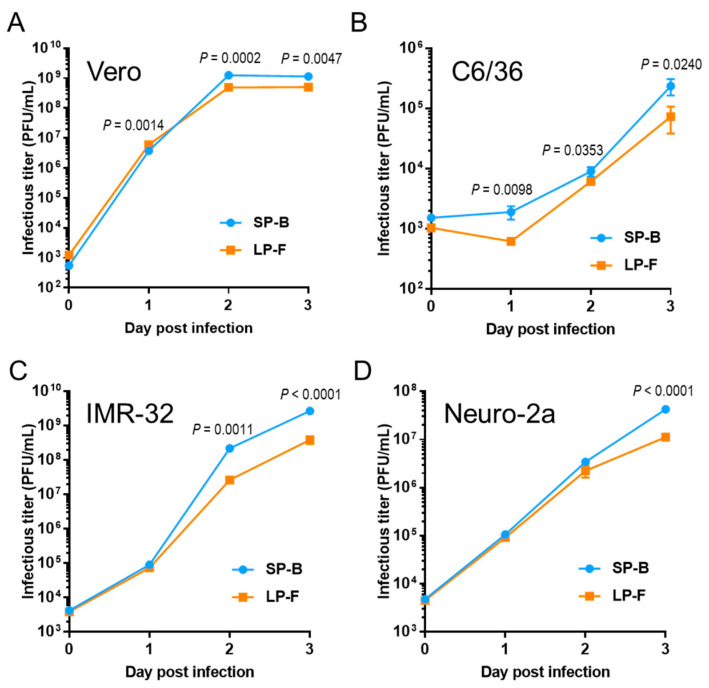
Growth kinetics of OR393 SP-B and LP-F clones in Vero (**A**), C6/36 (**B**), IMR-32 (**C**), and Neuro-2a (**D**). Cells were infected at MOI of 0.05 (Vero, IMR-32, and Neuro-2a) or 0.01 (C6/36). Values: means ± standard deviation from three independent inoculations. Significance was analyzed using Student’s *t*-test. *p*-values are also indicated.

We selected the SP clone SP-B (GenBank accession No. LC802098) and the LP clone LP-F for further characterization in vitro. The growth rate of SP-B was slightly higher than that of LP-F in Vero, mosquito C6/36, human neuroblastoma IMR-32, and mouse neuroblastoma Neuro-2a cells (Figure 2).

### 3.3. Glycosylation Status of the E Protein of Small-Sized and Large-Sized Plaque WNV_KUN_ Clones

**Figure 3:**
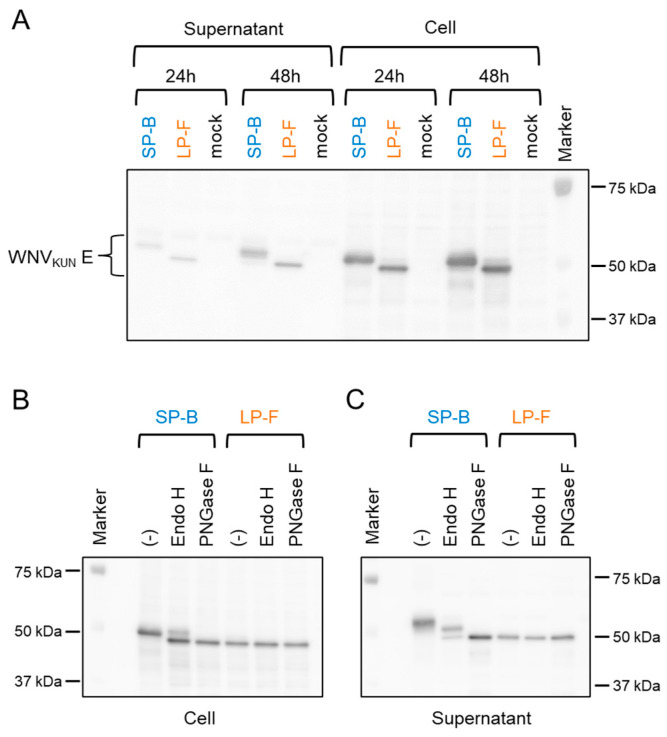
Immunoblot analysis of culture supernatant and cell lysate of SP-B- and LP-F-infected Vero cells. (**A**) WNV_KUN_ E protein in the samples was detected with an anti-WNV E antibody GTX132052. (**B**,**C**) Cell lysates (**B**) and culture supernatants (**C**) were treated with endoglycosidase H (Endo H) and peptide N-glycosidase F (PNGase F) before loading onto an SDS-PAGE gel. WNV_KUN_ E protein in the samples was detected with the anti-WNV E antibody. Mock indicates mock-inoculated samples. (-) indicates non-glycosidase reaction control. Markers indicate molecular weight markers.

Asn at position E^154^ is an N-linked glycosylation site in the WNV E protein, and the amino acid motif from E^154^ to E^156^ (Asn-Tyr-Ser) is critical for this modification. E^156^ was Ser in the SP clones and Phe in the LP clones (Table 1). SDS-PAGE and Immunoblot analyses showed that the E protein of SP-B migrated slower than that of LP-F, suggesting that the difference in the migration rate of the E protein between SP-B and LP-F was due to the glycosylation pattern at position E^154^ (Figure 3A). To confirm the effect of glycosylation of the E protein on the mobility shift, the cell lysate and supernatant samples were treated with two glycosidases, Endo H and PNGase F (Figure 3B,C). PNGase F removes almost all types of N-linked (Asn-linked) glycosylation, while Endo H removes only high-mannose and some hybrid types of N-linked carbohydrates. SP-B E protein treated with the enzymes migrated faster than the untreated SP-B E protein. In contrast, there was no change in the migration rate of the LP-F E protein after treatment with the enzymes. Furthermore, the mobility of the PNGase F-treated SP-B E protein was similar to that of the LP-F E protein. These data indicated that Ser at position E^156^ is involved in the glycosylation of E in the SP-B clone.

### 3.4. Mutations at E^156^ and E^332^ of the WNV_KUN_ LP Clone Affected Plaque Formation and Growth In Vitro

**Figure 4:**
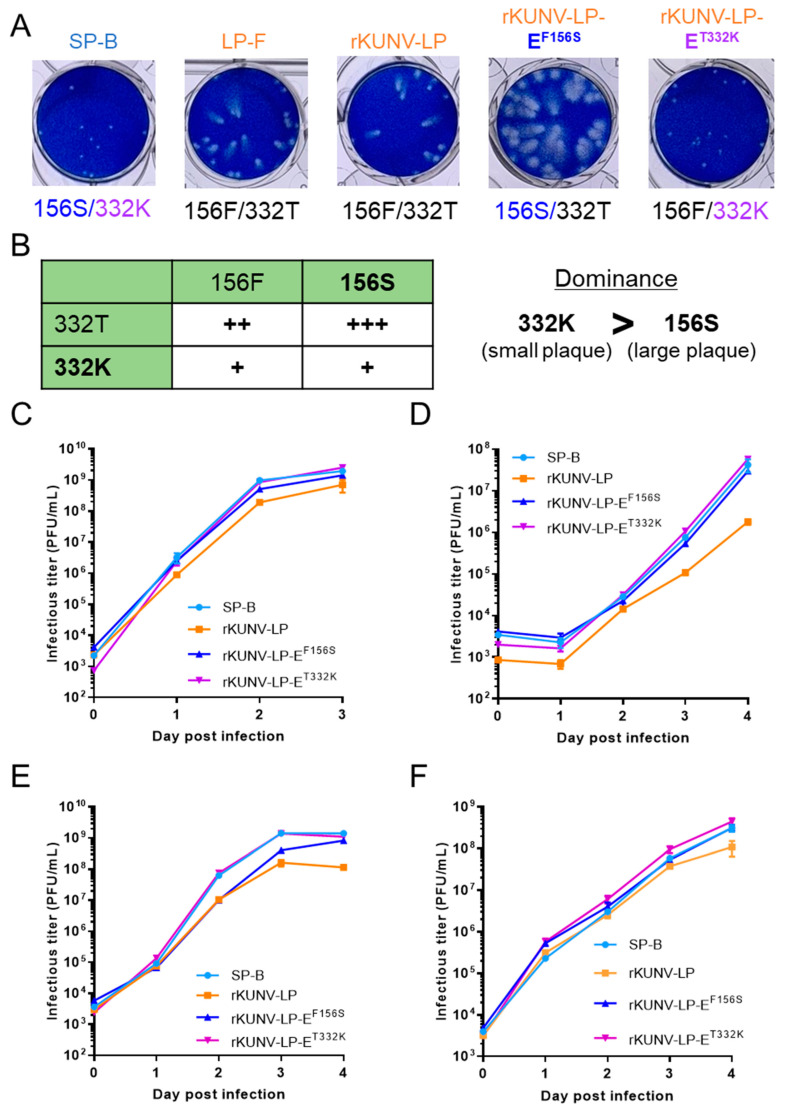
Growth properties of recombinant WNV_KUN_ rKUNV-LP, rKUNV-LP-E^F156S^, and rKUNV-LP-E^T332K^. (**A**) Plaque phenotypes of recombinant WNV_KUN_ viruses in Vero cells. Five days post-inoculation, the cells were fixed and stained. (**B**) Summary of the relationship between plaque size and the amino acid residues E^156^ and E^332^. +, small size; ++, medium size; +++, large size. (**C**–**F**) Growth kinetics of recombinant WNV_KUN_ strains in Vero (**C**), C6/36 (**D**), IMR-32 (**E**), and Neuro-2a (**F**). Cells were infected at MOI of 0.05 (Vero, IMR-32, and Neuro-2a) or 0.01 (C6/36). Values: means ± standard deviation from three independent inoculations. Significance was analyzed using Student’s *t*-test. *p*-values (rKUNV-LP vs. rKUNV-LP-E^F156S^ and rKUNV-LP vs. rKUNV-LP-E^T332K^) are shown in Supplementary Table S2.

To further investigate the role of the amino acid variations found in SP and LP in vitro and in vivo, a reverse-genetics system for the WNV_KUN_ LP-F clone was established (Figure S1). The plaques formed by the recombinant LP clone (mean diameter ± SD: 1.06 ± 0.124 mm) closely resembled those of the LP-F clone (1.06 ± 0.145 mm) in Vero cells (Figure 4A). Using this system, two mutant WNV_KUN_ LP clones, rKUNV-LP-E^F156S^ and rKUNV-LP-E^T332K^, were generated (Figure 4A and Figure S1). The plaques formed by rKUNV-LP-E^F156S^ (1.74 ± 0.226 mm) were larger than those formed by SP-B (0.71 ± 0.081 mm) and LP-F (Figure 4A). rKUNV-LP-E^T332K^ formed plaques whose size (0.76 ± 0.087 mm) was similar to that of SP-B. The plaque size of the E^156S/332T^ virus (rKUNV-LP-E^F156S^) was larger than that of the E^156F/332T^ virus (LP virus), but the size of the E^156S/332K^ virus (SP-B) was equivalent to that of the E^156F/332K^ virus (rKUNV-LP-E^T332K^). The plaques formed by the E^332K^ viruses (SP-B and rKUNV-LP-E^T332K^) were smaller than those formed by the E^332T^ viruses (LP clones and rKUNV-LP-E^F156S^). These results indicate that the amino acid residue of E^332^ was dominant to that of E^156^ in regulating the plaque size formed by LP-F, and, therefore, the plaque size is mainly driven by E^332^ in SP and LP variants (Figure 4B). The growth rate of rKUNV-LP-E^F156S^ and rKUNV-LP-E^T332K^ resembled SP-B in Vero, C6/36, and Neuro-2A cells (Figure 4C). SP-B and rKUNV-LP-E^T332K^ grew faster than rKUNV-LP and rKUNV-LP-E^F156S^ in IMR-32 cells.

### 3.5. Virulence of the WNV_KUN_ SP and LP Clones and Recombinant WNV_KUN_ Mutants in Mice

**Figure 5:**
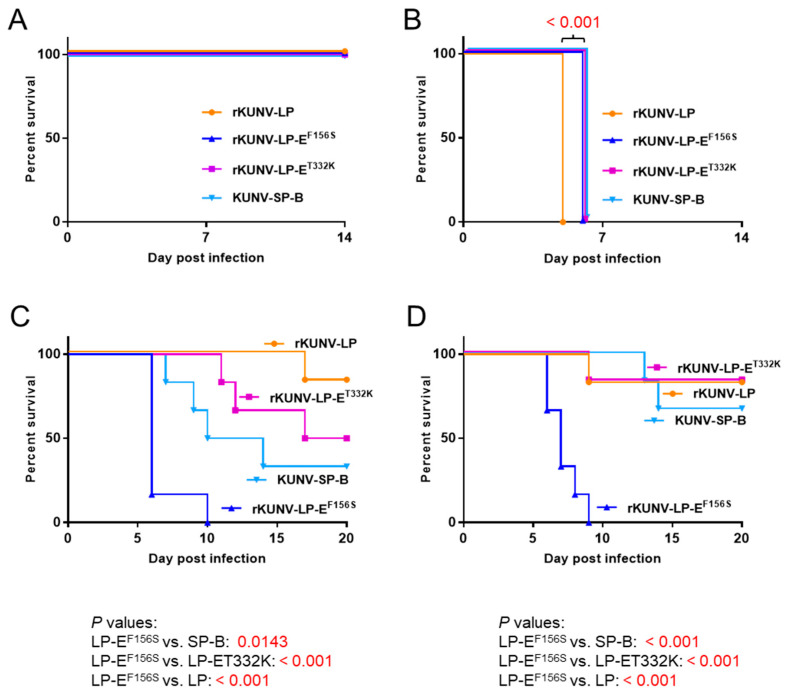
Neurovirulence and neuroinvasiveness of the recombinant WNV_KUN_ strains. (**A**,**B**) Survival curves of mice inoculated i.c. with 3 × 10^2^ PFU (**A**) and 3 × 10^3^ PFU (**B**) of rKUNV-LP (*n* = 6), rKUNV-LP-E^F156S^ (*n* = 6), rKUNV-LP-E^T332K^ (*n* = 6), or KUNV-SP-B (*n* = 6). (**C**,**D**) Survival curves of mice intraperitoneally inoculated with 5 × 10^4^ PFU or 5 × 10^5^ PFU of rKUNV-LP (*n* = 6), rKUNV-LP-E^F156S^ (*n* = 6), rKUNV-LP-E^T332K^ (*n* = 6), or KUNV-SP-B (*n* = 6). Significant *P* values determined by log-rank (Mantel–Cox) tests are also indicated. No deaths occurred in the saline-inoculated group.

We examined the neurovirulence and neuroinvasiveness of the SP, LP, and recombinant mutants in mice. Mice were infected i.c. with SP-B, rKUNV-LP, rKUNV-LP-E^F156S^, or rKUNV-LP-E^T332K^. All mice inoculated with 3 × 10^2^ PFU of the viruses survived (Figure 5A). In the 3 × 10^3^ PFU-inoculated groups, all mice inoculated with rKUNV-LP died at 5 days post-infection, whereas all mice inoculated with SP-B, rKUNV-LP-E^F156S^, or rKUNV-LP-E^T332K^ died at 6 days post-infection (Figure 5B).

Mice were also infected i.p. with the four viruses. In the group infected with 5 × 10^4^ PFU, one (16.7%), three (50%), and four (66.7%) out of six mice inoculated with rKUNV-LP, rKUNV-LP-E^T332K^, and SP-B, respectively, died within the observation period, whereas all rKUNV-LP-E^F156S^-infected mice died by 10 days post-infection (Figure 5C). In the group infected with 5 × 10^5^ PFU, at least four (66.7%) of the six mice inoculated with rKUNV-LP, rKUNV-LP-E^T332K^, or SP-B survived throughout the observation period, but all mice inoculated with rKUNV-LP-E^F156S^ died within 9 days post-infection (Figure 5D).

### 3.6. Growth of the WNV_KUN_ SP and LP Clones and Recombinant WNV_KUN_ Mutants in Mice

**Figure 6:**
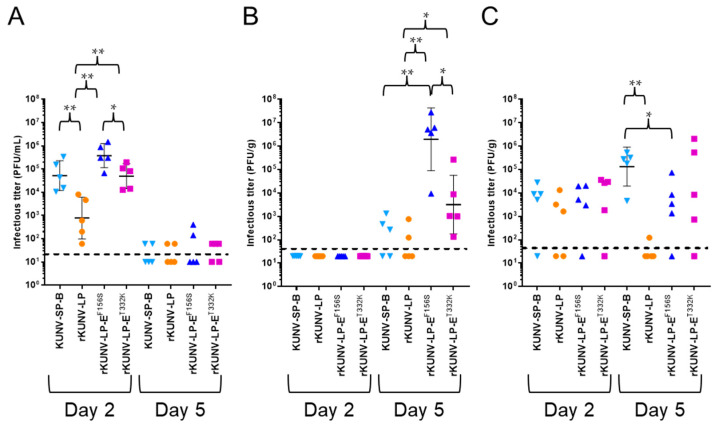
Levels of infectious virus at 2 and 5 days after inoculation of WNV_KUN_-infected mice. Mice inoculated i.p. with 1 × 10^4^ PFU of KUNV-SP-B (*n* = 5), rKUNV-LP (*n* = 5), rKUNV-LP-E^F156S^ (*n* = 5), or rKUNV-LP-E^T332K^ (*n* = 5) were euthanized at 2 or 5 days after inoculation, and serum (**A**), brain (**B**), and spleen (**C**) samples were collected. Sera and tissue homogenates were used to quantify the infectious virus titer (PFU/mL or g). Dotted line: detection limit. Geometric mean titers and geometric standard deviations are indicated by horizontal bars. Significance was analyzed using the Mann–Whitney U test (* *p* < 0.05, ** *p* < 0.01).

Infectious viruses and viral RNA levels were investigated in mice inoculated i.p. with the recombinant viruses (Figure 6). Two days after inoculation, infectious viruses were detected in most serum and spleen samples, although no infectious viruses were detected in the brains of any of the four groups. High levels of viremia were observed in the sera of mice inoculated with rKUNV-LP-E^F156S^, rKUNV-LP-E^T332K^, and SP-B strains (Figure 6A). The levels of viral RNA in the serum samples were also significantly higher in rKUNV-LP-E^F156S^-, rKUNV-LP-E^T332K^-, and SP-B-inoculated mice than in rKUNV-LP-inoculated animals (Figure S2). In spleen samples, no clear differences in infectious titers were observed among the strains (Figure 6C). Five days after inoculation, the number of infectious viruses decreased and was not observed in half of the serum samples from any of the four groups (Figure 6A). In the brain samples, significantly higher levels of the infectious virus were detected in the rKUNV-LP-E^F156S^-inoculated group (Figure 6B). In contrast, the level of infectious viruses in SP-B-infected mice was higher than that in rKUNV-LP-E^F156S^-infected mice (Figure 6C). Samples from rKUNV-LP-infected mice showed lower levels of viremia and infectious viruses than those from other virus-infected groups.

### 3.7. Genomic Stability of the E^156^ and E^332^ Mutations in the Recombinant WNV_KUN_ Mutants

**Figure 7:**
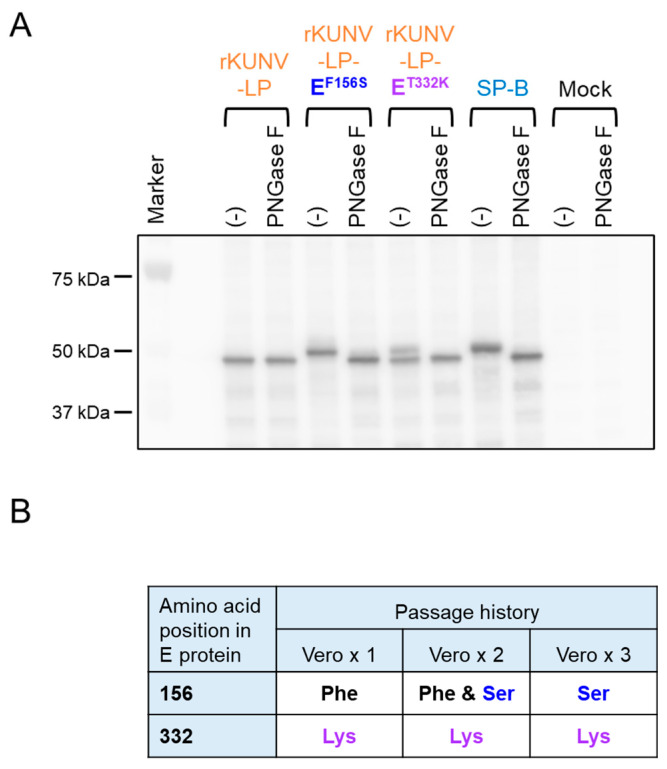
(**A**) Immunoblot analysis of cell lysates from recombinant WNV_KUN_-infected Vero cells. Cells were collected 48 h after virus inoculation and lysed. The cell lysate was treated with PNGase F before loading onto SDS-PAGE gel. WNV_KUN_ E protein in the samples was detected using an anti-WNV E antibody. SP-B-infected cell samples were used as N-glycosylation-positive controls. Mock indicates mock-infected Vero cell lysates. (−): no PNGase F-treated lysate. (**B**) Amino acid residues at E^156^ and E^332^ in rKUNV-LP-E^T332K^ viruses passaged repeatedly in Vero cells.

We confirmed the N-glycosylation of recombinant WNV_KUN_ E proteins in Vero cells via immunoblot analysis (Figure 7A). The E protein of rKUNV-LP-E^F156S^, similar to SP-B, migrated more slowly than rKUNV-LP, and the mobility of the PNGase F-treated rKUNV-LP-E^F156S^ E protein was similar to that of the rKUNV-LP E protein, indicating that the rKUNV-LP-E^F156S^ E protein was glycosylated in Vero cells. However, two different migration signals, rKUNV-LP-like and SP-B-like (slow) patterns, were observed in rKUNV-LP-E^T332K^-infected cell samples, and the SP-B-like pattern disappeared after treatment with PNGase F. Nucleotide sequences at sites E^156^ and E^332^ were determined in rKUNV-LP-E^T332K^ passaged once, twice, and three times in Vero cells (Figure 7B). No mutation at E^332^ was observed in the three viruses, whereas the amino acid residue at E^156^ was partially changed from Phe to Ser (from U to C at nucleotide position 1433) in the twice-passaged virus and completely changed in the three-times-passaged virus. We also examined the nucleotide sequences of sites E^156^ and E^332^ in day 5 mouse brain samples used for the growth analysis shown in Figure 6 (Table S3). The amino acid residue at E^156^ of the virus detected in the rKUNV-LP-E^T332K^-infected mouse brains was Ser (C at nucleotide position 1433) in all three samples examined. Partial amino acid changes from Phe to Ser were also observed in the brain samples of mice infected with rKUNV-LP.

## 4. Discussion

In this study, we investigated the characteristics of the WNV_KUN_ OR393 strain, which was isolated from *Culex* mosquitoes in Australia in the 1970s, to evaluate the possibility of using this virus as a candidate backbone to develop a live-attenuated WNV vaccine. However, the original stock of the virus was mixed with two substrains (LP and SP) with different plaque-formation abilities in Vero cells.

We obtained several clones of LP and SP, and their nucleotide sequences indicated that the two amino acid residues at E^156^ and E^332^ are involved in the plaque phenotype. Adams et al. showed that the amino acid residue of E^156^ of OR393 was phenylalanine [19], suggesting that the mutation at E^156^ may have occurred during the process of virus passage, although the exact passage history is unknown. As mentioned later, we proved in this study that the mutation at E^156^ occurs easily by passaging rKUNV-LP-E^T332K^ in Vero cells (Figure 7). Analysis using recombinant WNV_KUN_ mutants clearly showed that the E^156S^ virus formed larger plaques than the E^156F^ virus when the residue E^332^ was Thr. The WNV E protein is composed of three structural domains: I, II, and III (EDI, EDII, and EDIII) [31]. E^156^ is located on the N-glycosylation motif (Asn-Tyr-Ser) in the EDI, and this residue influences the N-glycosylation of the Asn residue at E^154^, suggesting that glycosylation is associated with the plaque morphology of WNV_KUN_. We confirmed that the E protein of SP-B is N-glycosylated, whereas that of LP-F is not. However, E^332K^ potently decreased the plaque size, regardless of the residue at position E^156^. These results indicate that the residue of E^332^ is a dominant determinant of plaque size. The LP strains (LP-F and rKUNV-LP) grew slower than the other strains, indicating that the combination of E^156F^ and E^332T^ decreased virus growth in cultured cells. The threonine residue at E^332^ is conserved among WNV_KUN_, and the Lys residue at this position is unique to SP-B (Figure S3). Although the reason why E^332K^ emerged during passaging in the mouse brain and Vero cells remains unknown, our data imply that E^332K^ may be advantageous for growth in Vero cells or the mouse brain when the residue in E^156^ is phenylalanine rather than serine. Our plaque and growth analyses in Vero cells also demonstrated that the growth rate is not necessarily correlated with plaque size in Vero cells among OR393 substrains.

The survival curves of the four WNV_KUN_ strains in the i.c. inoculation experiments were similar, and all infected mice died 6 days post-infection, suggesting that these viruses have equivalent neurovirulence in mice. However, all rKUNV-LP-inoculated mice died 5 days post-infection, whereas mice inoculated with the other strains died 6 days post-infection in the 3 × 10^3^ PFU/mouse group. Moreover, in the 1.5 × 10^4^ PFU/mouse inoculation, all rKUNV-LP-inoculated mice died at 5 days post-infection; however, most of the mice inoculated with the other strains died at 6 days post-infection (Figure S4). These results were unexpected because previous reports have revealed that E^156S^ in WNV is a virulent type, but E^156F^ is not. An analysis using chimeric viruses between the virulent WNV and non-pathogenic WNV_KUN_ also suggested that not only E^156^ but also other regions of the E protein are important for the pathogenicity of WNV [25]. E^332^ is located in EDIII, which forms an immunoglobulin-like domain that is thought to play a crucial role in receptor binding and viral attachment to the cell surface (Figure S5) [32]. Mutations in EDIII result in altered virulence, suggesting that this domain is involved in viral pathogenesis [33,34].

In contrast, the results of i.p. inoculation indicated that rKUNV-LP-E^F156S^ exhibited a significantly higher neuroinvasive ability than the other three strains. Furthermore, the infectious virus levels in rKUNV-LP-E^F156S^-infected mouse brains were higher than those in mice infected with other viruses. These results demonstrated that the E^156S/332T^-type virus has the potential to increase the neuroinvasiveness of WNV_KUN_. Previous studies have indicated the importance of N-glycosylation of E protein in the neurovirulence and neuroinvasiveness of WNV in mammalian hosts [8,9,21,22,23,25]. Glycosylation influences virus binding to cell surface attachment factors and the infectivity of WNV [24]. WNV strains containing N-glycosylation at E^154^ use DC-SIGN, a C-type lectin present on the surface of dendritic cells, as an attachment factor to enhance infection compared with non-glycosylated strains [35]. DC-SIGNR also promotes WNV infection more efficiently than DC-SIGN in mammalian cells, and this effect is dependent on N-glycosylation [36]. These previous findings may help understand the basis of the increased growth and pathogenicity of the E^156S/332T^-type WNV_KUN_ strains. In contrast, E^156S/332K^-type SP-B resulted in lower viremia levels and neuroinvasiveness than E^156S/332T^-type rKUNV-LP-E^F156S^ in mice. The E^156F/332T^-type rKUNV-LP showed the lowest neuroinvasiveness and infectious virus levels in the serum, brain, and spleen of mice, implying that, in contrast to the results of the neurovirulence analysis, the combination of E^156F^ and E^332T^ may be negatively associated with neuroinvasiveness in mice. Thus, our data suggest that the amino acid residues at E^156^ and E^332^ of WNV_KUN_ play different roles in neurovirulence and neuroinvasiveness. Further comprehensive analyses are required to understand the mechanisms of action of these residues. Generally, pathogenic WNV produces large plaques, which are indicative of rapid cell proliferation. However, our data demonstrated that the plaque size formed by infection with WNV cannot be used as an indicator of its virulence [37]. Our results also showed that low-pathogenic classical strains of WNV_KUN_ could be easily transformed into highly pathogenic viruses by only a few amino acid substitutions in the E protein. The outbreak of WNV in horses in Australia in the 2010s may have been caused by several mutations in the WNV_KUN_ genome that made the virus more virulent [18]. It is important to continue to analyze the genome of the virus and pay attention to the genome sequences of WNV_KUN_.

Our data in Figure 7 and Table S3 show that the Phe residue at E^156^ in rKUNV-LP-E^T332K^ was rapidly replaced with Ser in Vero cells and in mice. This implies that rKUNV-LP-E^T332K^ is changed to the SP (E^156S/332K^) virus by passaging in these cells. The plaque sizes formed by SP-B and rKUNV-LP-E^T332K^ were so close that it was difficult to discern the viruses using plaque morphology (Figure 4A). These data also raise the possibility that the amino acid residue at E^156^ may be partially altered in in vitro growth analysis and virulence analysis in mice. The neuroinvasiveness of rKUNV-LP-E^T332K^ was intermediate compared to that of rKUNV-LP and SP-B (Figure 5C). However, the amino acid residue of E^332^ of rKUNV-LP-E^T332K^ was changed from Lys to Thr in mouse brain samples inoculated with the virus (Table S3), suggesting that the neuroinvasive ability of rKUNV-LP-E^T332K^ may be associated with a Phe-to-Ser substitution at E^156^ of the virus in mice.

This study showed that LP-F exhibited lower neuroinvasiveness than SP-B. Furthermore, the neuroinvasiveness of LP-F was lower than that of the two recombinant WNV_KUN_ viruses produced in this study. An analysis of the attenuated JE vaccine strain SA 14-14-2 revealed that 10 amino acids in the E protein are involved in the attenuation of the parental JEV strain SA 14 [38,39]. WNV is genetically close to JEV, and 9 of the 10 mutation sites involved in JEV attenuation are conserved in WNV. Chimeri-Vax-WN02, a live chimeric WNV vaccine based on the YFV vaccine strain, also incorporates three of the nine putative attenuating mutations [40]. The LP strain of WNV_KUN_ OR393 exhibited weak neuroinvasiveness; therefore, the LP strain could be more easily attenuated than the NY99 strain. A safer, live-attenuated WNV vaccine could be developed by introducing putative attenuating mutations into the LP strain; however, it must be kept in mind that the genomic sequence of LP may not be necessarily stable in host cells.

## Figures and Tables

**Table 1:** Nucleotide and amino acid variations in small- and large-plaque clones isolated from the WNV_KUN_ OR393 strain.

		Small-Plaque Clones								Large-Plaque Clones					
		SP-A		SP-B		SP-C		SP-D		LP-E		LP-F		LP-G	
Region	NT Position	NT	AA	NT	AA	NT	AA	NT	AA	NT	AA	NT	AA	NT	AA
E	1433	C	Ser	C	Ser	C	Ser	C	Ser	U	Phe	U	Phe	U	Phe
	1961	A	Lys	A	Lys	A	Lys	A	Lys	C	Thr	C	Thr	C	Thr
	2169	A	Gly	A	Gly	ND	ND	ND	ND	A	Gly	G	Gly	ND	ND
	2271	U	Phe	U	Phe	ND	ND	ND	ND	U	Phe	C	Phe	ND	ND
NS5	8613	U	Tyr	C	Tyr	ND	ND	ND	ND	C	Tyr	C	Tyr	ND	ND
3′NCR	10,484	A	-	A	-	ND	ND	ND	ND	G	-	A	-	ND	ND

NT, nucleotide; AA, amino acid; ND, not determined.

## Data Availability

The data presented in this study are available upon request from the corresponding author.

## Supplementary Materials

The following supporting information can be downloaded at: https://www.mdpi.com/article/10.3390/v16081237/s1, Figure S1: Schematic representation of the recombinant WNV_KUN_ production. Figure S2: Levels of genomic copy number at 2 and 5 days after inoculation of WNV_KUN_-infected mice. Figure S3: Comparison of the amino acid sequences of the E protein (501 residues) in the WNVKUN and L1 WNV strains NY99. Figure S4: Neurovirulence of the WNV_KUN_ strains. Figure S5: Structure of West Nile virus E protein (PDB ID: 2I69). Table S1: List of oligonucleotides used in this study. Table S2: *p* values in Figure 4.
