# Correction: Tajima et al. Amino Acids at Positions 156 and 332 in the E Protein of the West Nile Virus Subtype Kunjin Virus Classical Strain OR393 Are Involved in Plaque Size, Growth, and Pathogenicity in Mice. *Viruses* 2024, *16*, 1237

**DOI:** 10.3390/v16111736

**Published:** 2024-11-05

**Authors:** Shigeru Tajima, Hideki Ebihara, Chang-Kweng Lim

**Affiliations:** Department of Virology I, National Institute of Infectious Diseases, 1-23-1 Toyama, Shinjuku, Tokyo 162-8640, Japan

Missing Table

In the original publication [1], there was a mistake in the Supplementary Materials file as published. Table S3 has been removed from the file. The corrected Supplementary Table S3 “Amino acid residues at positions E^156^ and E^332^ in viruses recovered from brain samples in Figure 6” is presented below, and the table has been added in the file.

**Table S3:** Amino acid residues at positions E^156^ and E^332^ in viruses recovered from brain samples in Figure 6.

Day 5 mouse brain infected with:	Amino acid at position in E	
	156	332
SP-B.2	Ser	Lys
SP-B.3	Ser	Lys
SP-B.5	Ser	Lys
rKUNV-LP.2	Phe	Thr
rKUNV-LP.3	Phe and Ser	Thr
rKUNV-LP-E^F156S^.1	Ser	Thr
rKUNV-LP-E^F156S^.2	Ser	Thr
rKUNV-LP-E^F156S^.3	Ser	Thr
rKUNV-LP-E^F156S^.4	Ser	Thr
rKUNV-LP-E^F156S^.5	Ser	Thr
rKUNV-LP-E^T332K^.1	Ser	Lys
rKUNV-LP-E^T332K^.2	Ser	Lys
rKUNV-LP-E^T332K^.3	Ser	Lys

Text Correction

A correction has been made to “Supplementary Materials” of the main text, It should be read:

**Supplementary Materials:** The following supporting information can be downloaded at: https://www.mdpi.com/article/10.3390/v16081237/s1, Figure S1: Schematic representation of the recombinant WNV_KUN_ production. Figure S2: Levels of genomic copy number at 2 and 5 days after inoculation of WNV_KUN_-infected mice. Figure S3: Comparison of the amino acid sequences of the E protein (501 residues) in the WNV_KUN_ and L1 WNV strains NY99. Figure S4: Neurovirulence of the WNV_KUN_ strains. Figure S5: Structure of West Nile virus E protein (PDB ID: 2I69). Table S1: List of oligonucleotides used in this study. Table S2: *p* values in Figure 4. Table S3: Amino acid residues at positions E^156^ and E^332^ in viruses recovered from brain samples in Figure 6.

The authors state that the scientific conclusions are unaffected. This correction was approved by the Academic Editor. The original publication has also been updated.
