# Supplementary material for: Amino Acids at Positions 156 and 332 in the E Protein of the West Nile Virus Subtype Kunjin Virus Classical Strain OR393 Are Involved in Plaque Size, Growth, and Pathogenicity in Mice
Source: Viruses. 2024 Aug 1;16(8):1237. doi: 10.3390/v16081237 (PMC11359920; doi:10.3390/v16081237)
Supplement: Supplementary file 1 [file viruses-16-01237-s001.zip › viruses-3014130-supplementary.pdf]

Figure S1

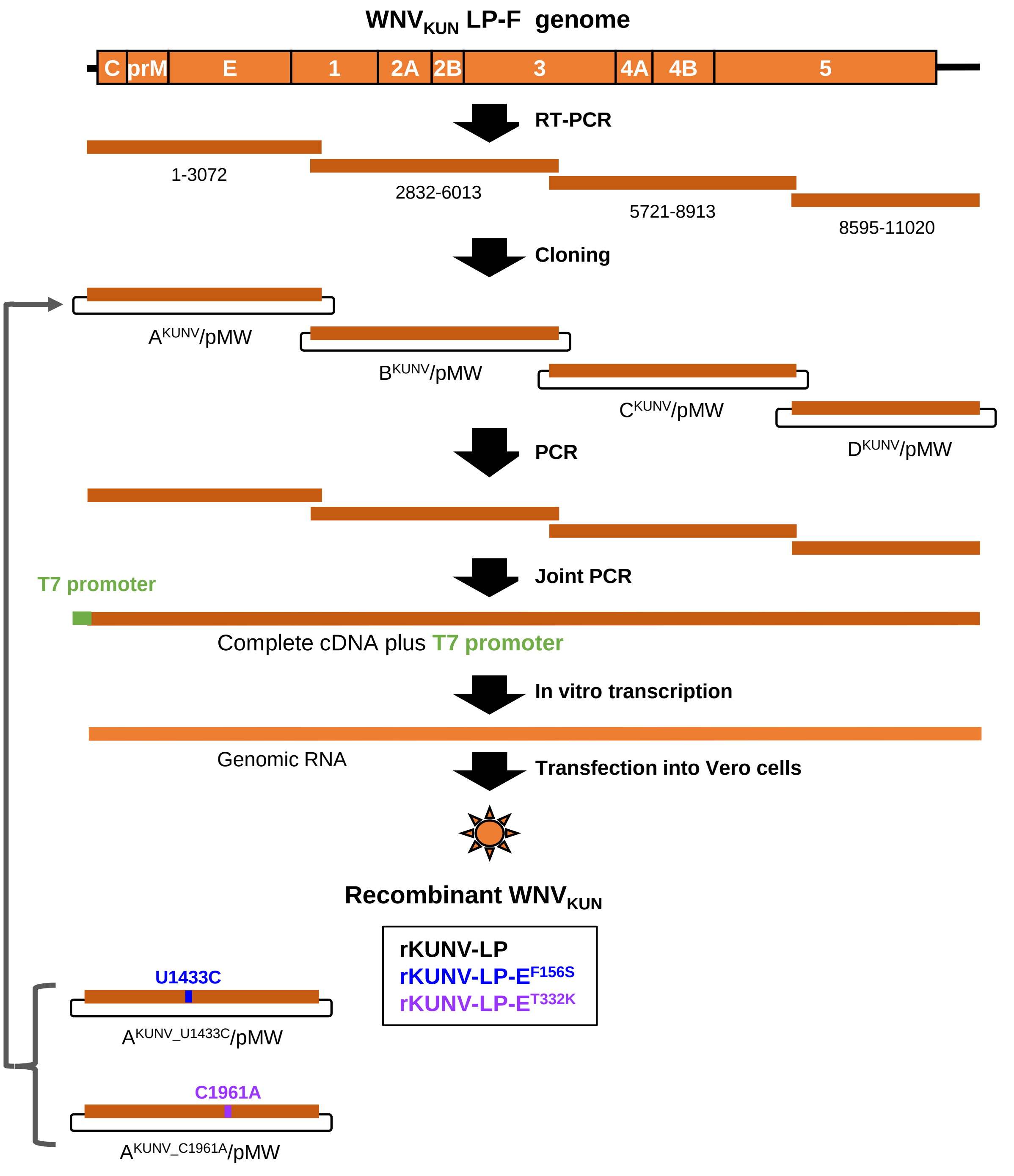

Figure S1. Schematic representation of the recombinant WNV$_{KUN}$ production. The method for the production is described in Materials and Methods section in detail.

Figure S2

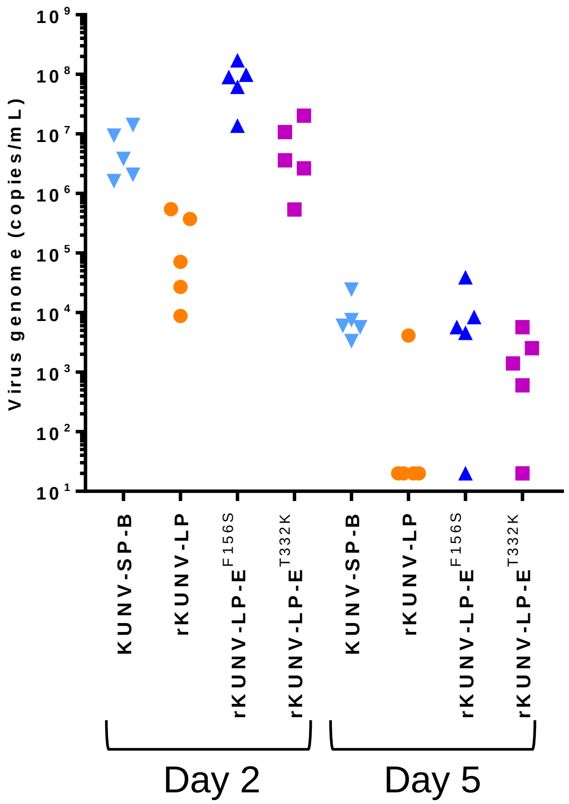

Figure S2. Levels of genomic copy number at 2 and 5 days after inoculation of WNV_{KUN}-infected mice. Mice inoculated i.p. with 1 × 10^4 PFU of KUNV-SP-B (*n* = 5), rKUNV-LP (*n* = 5), rKUNV-LP-$E^{F\ 156S}$ (*n* = 5), or rKUNV-LP-E^{T332K} (*n* = 5) were euthanized at two or five days after inoculation, and samples were collected. Sera were used to quantify the genomic copy number (PFU/mL) as described in the Materials and Methods section.

Figure S3

H
Virulence
L

```
NY99       1 FNCLGMSNRDFLEGVSGATWVDLVLEGDSCVTIMSKDKPTIDVKMMNMEAANLAEVRSYCYLATVSDLSTKAACPTMGEAHNDKRADPAFVCRQGVVDRGWGNGCGLFGK 110
SP-B       1 FNCLGMSNRDFLEGVSGATWVDLVLEGDSCVTIMSKDKPTIDVKMMNMEAANLAEVRSYCYLATVSELSTKAACPTMGEAHNDKRADPSFVCKQGVVDRGWGNGCGLFGK 110
LP-F       1 FNCLGMSNRDFLEGVSGATWVDLVLEGDSCVTIMSKDKPTIDVKMMNMEAANLAEVRSYCYLATVSELSTKAACPTMGEAHNDKRADPSFVCKQGVVDRGWGNGCGLFGK 110
NSW2012    1 FNCLGMSNRDFLEGVSGATWVDLVLEGDSCVTIMSKDKPTIDVRMMNMEAANLAEVRSYCYLATVSELSTKAACPTMGEAHNDKRADPSFVCKQGVVDRGWGNGCGLFGK 110
NSW2011    1 FNCLGMSNRDFLEGVSGATWVDLVLEGDSCVTIMSKDKPTIDVRMMNMEAANLAEVRSYCYLATVSELSTKAACPTMGEAHNDKRADPSFVCKQGVVDRGWGNGCGLFGK 110
GU0631     1 FNCLGMSNRDFLEGVSGATWVDLVLEGDSCVTIMSKDKPTIDVKMMNMEAANLAEVRSYCYLATVSELSTKAACPTMGEAHNDKRADPSFVCKQGVVDRGWGNGCGLFGK 110
Boort      1 FNCLGMSNRDFLEGVSGATWVDLVLEGDSCVTIMSKDKPTIDVKMMNMEAANLAEVRSYCYLATVSELSTKAACPTMGEAHNDKRADPSFVCKQGVVDRGWGNGCGLFGK 110
GU1009     1 FNCLGMSNRDFLEGVSGATWVDLVLEGDSCVTIMSKDKPTIDVRMMNMEAANLAEVRSYCYLATVSELSTKAACPTMGEAHNDKRADPSFVCKQGVVDRGWGNGCGLFGK 110
K6453      1 FNCLGMSNRDFLEGVSGATWVDLVLEGDSCVTIMSKDKPTIDVKMMNMEAANLAEVRSYCYLATVSELSTKAACPTMGEAHNDKRADPSFVCKQGVVDRGWGNGCGLFGK 110
P9974      1 FNCLGMSNRDFLEGVSGATWVDLVLEGDSCVTIMSKDKPTIDVRMMNMEAANLAEVRSYCYLATVSELSTKAACPTMGEAHNDKRADPSFVCKQGVVDRGWGNGCGLFGK 110
K74015     1 FNCLGMSNRDFLEGVSGATWVDLVLEGDSCVTIMSKDKPTIDVRMMNMEAANLAEVRSYCYLATVSELSTKAACPTMGEAHNDKRADPSFVCKQGVVDRGWGNGCGLFGK 110
K68967     1 FNCLGMSNRDFLEGVSGATWVDLVLEGDSCVTIMSKDKPTIDVRMMNMEAANLAEVRSYCYLATVSELSTKAACPTMGEAHNDKRADPSFVCKQGVVDRGWGNGCGLFGK 110

NY99     111 GSIDTCAKFACSTKAIGRTILKENIKYEVAIFVHGPTTVESHGNYSTQVGATQAGRLSITPAAPSYTLKLGEYGEVTVDCEPRSGIDTNAYYVMTVGTKTFLVHREWFMD 220
SP-B     111 GSIDTCAKFACSTKATGRTILKENIKYEVAIFVHGPTTVESHGNYSTQTGAAQAGRFSITPAAPSYTLKLGEYGEVTVDCEPRSGIDTSAYYVMTVGTKTFLVHREWFMD 220
LP-F     111 GSIDTCAKFACSTKATGRTILKENIKYEVAIFVHGPTTVESHGNYFTQTGAAQAGRFSITPAAPSYTLKLGEYGEVTVDCEPRSGIDTSAYYVMTVGTKTFLVHREWFMD 220
NSW2012  111 GSIDTCAKFACSTKATGRTILKENIKYEVAIFVHGPTTVESHGNYSTQTGAAQAGRFSITPAAPSYTLKLGEYGEVTVDCEPRSGIDTSAYYVMTVGTKTFLVHREWFMD 220
NSW2011  111 GSIDTCAKFACSTKATGRTILKENIKYEVAIFVHG [illegible] NYSTQTGAAQAGRFSITPAAPSYTLKLGEYGEVTVDCEPRSGIDTSAYYVMTVGTKTFLVHREWFMD 220
GU0631   111 GSIDTCAKFACSTKATGRTILKENIKYEVAIFVHG   156   NYSTQTGAAQAGRFSITPAAPSYTLKLGEYGEVTVDCEPRSGIDTSAYYVMTVGTKTFLVHREWFMD 220
Boort    111 GSIDTCAKFACSTKATGRTILKENIKYEVAIFVHGPTTVESHGNYSTQTGAAQAGRFSITPAAPSYTLKLGEYGEVTVDCEPRSGIDTSAYYVMTVGTKTFLVHREWFMD 220
GU1009   111 GSIDTCAKFACSTKATGRTILKENIKYEVAIFVHGPTTVESHGNYSTQTGAAQAGRFSITPAAPSYTLKLGEYGEVTVDCEPRSGIDTSAYYVMTVGTKTFLVHREWFMD 220
K6453    111 GSIDTCAKFACSTKATGRTILKENIKYEVAIFVHGPTTVESHGNYSTQTGAAQAGRFSITPAAPSYTLKLGEYGEVTVDCEPRSGIDTSAYYVMTVGTKTFLVHREWFMD 220
P9974    111 GSIDTCAKFACSTKATGRTILKENIKYEVAIFVHGPTTVESHGNYSTQTGAAQAGRFSITPAAPSYTLKLGEYGEVTVDCEPRSGIDTSAYYVMTVGTKTFLVHREWFMD 220
K74015   111 GSIDTCAKFACSTKATGRTILKENIKYEVAIFVHGPTTVESHGNYSTQTGAAQAGRFSITPAAPSYTLKLGEYGEVTVDCEPRSGIDTSAYYVMTVGTKTFLVHREWFMD 220
K68967   111 GSIDTCAKFACSTKATGRTILKENIKYEVAIFVHGPTTVESHGNYSTQTGAAQAGRFSITPAAPSYTLKLGEYGEVTVDCEPRSGIDTSAYYVMTVGTKTFLVHREWFMD 220

NY99     221 LNLPWSSAGSTVWRNRETLMEFEEPHATKQSVIALGSQEGALHQALAGAIPVEFSSNTVKLTSGHLKCRVKMEKLQLKGTTYGVCSKAFKFLGTPADTGHGTVVLELQYT 330
SP-B     221 LNLPWSSAESNVWRNRETLMEFEEPHATKQSVIALGSQEGALHQALAGAIPVEFSSNTVKLTSGHLKCRVKMEKLQLKGTTYGVCSKAFRFLGTPADTGHGTVVLELQYT 330
LP-F     221 LNLPWSSAESNVWRNRETLMEFEEPHATKQSVIALGSQEGALHQALAGAIPVEFSSNTVKLTSGHLKCRVKMEKLQLKGTTYGVCSKAFRFLGTPADTGHGTVVLELQYT 330
NSW2012  221 LNLPWSSAESNVWRNRETLMEFEEPHATKQSVIALGSQEGALHQALAGAIPVEFSSNTVKLTSGHLKCRVKMEKLQLKGTTYGVCSKAFRFLGTPADTGHGTVVLELQYT 330
NSW2011  221 LNLPWSSAESNVWRNRETLMEFEEPHATKQSVIALGSQEGALHQALAGAIPVEFSSNTVKLTSGHLKCRVKMEKLQLKGTTYGVCSKAFRFLGTPADTGHGTVVLELQYT 330
GU0631   221 LNLPWSSAESNVWRNRETLMEFEEPHATKQSVIALGSQEGALHQALAGAIPVEFSSNTVKLTSGHLKCRVKMEKLQLKGTTYGVCSKAFRFLGTPADTGHGTVVLELQYT 330
Boort    221 LNLPWSSAESNVWRNRETLMEFEEPHATKQSVIALGSQEGALHQALAGAIPVEFSSNTVKLTSGHLKCRVKMEKLQLKGTTYGICSKAFRFLGTPADTGHGTVVLELQYT 330
GU1009   221 LNLPWSSAESNVWRNRETLMEFEEPHATKQSVIALGSQEGALHQALAGAIPVEFSSNTVKLTSGHLKCRVKMEKLQLKGTTYGVCSKAFRFLGTPADTGHGTVVLELQYT 330
K6453    221 LNLPWSSAESNVWRNRETLMEFEEPHATKQSVIALGSQEGALHQALAGAIPVEFSSNTVKLTSGHLKCRVKMEKLQLKGTTYGVCSKAFRFLGTPADTGHGTVVMELQYT 330
P9974    221 LNLPWSSAESSVWRNRETLMEFEEPHATKQSVIALGSQEGALHQALAGAIPVEFSSNTVKLTSGHLKCRVKMEKLQLKGTTYGVCSKAFRFLGTPADTGHGTVVLELQYT 330
K74015   221 LNLPWSSAESNVWRNRETLMEFEEPHATKQSVIALGSQEGALHQALAGAIPVEFSSNTVKLTSGHLKCRVKMEKLQLKGTTYGVCSKAFRFLGTPADTGHGTVVLELQYT 330
K68967   221 LNLPWSSAESSVWRNRETLMEFEEPHATKQSVIALGSQEGALHQALAGAIPVEFSSNTVKLTSGHLKCRVKMEKLQLKGTTYGVCSKAFRFLGTPADTGHGTVVLELQYT 330

NY99     331 GTDGPCKVPISSVASLNDLTPVGRLVTVNPFVSVATANAKVLIELEPPFGDSYIVVGRGEQQINHHWHKSGSSIGKAFTTTLKGAQRLAALGDTAWDFGSVGGVFTSVGK 440
SP-B     331 GKDGPCKIPISSVASLNDLTPVGRLVTVNPFVSVSTANAKVLIELEPPFGDSYIVVGRGEQQINHHWHKSGSSIGKAFTATLKGAQRLAALGDTAWDFGSVGGVFTSVGK 440
LP-F     331 GTDGPCKIPISSVASLNDLTPVGRLVTVNPFVSVSTANAKVLIELEPPFGDSYIVVGRGEQQINHHWHKSGSSIGKAFTATLKGAQRLAALGDTAWDFGSVGGVFTSVGK 440
NSW2012  331 GTDGPCKIPISSVASLNDLTPVGRLVTVNPFVSVSTANAKVLIELEPPFGDSYIVVGRGEQQINHHWHKSGSSIGKAFTATLKGAQRLAALGDTAWDFGSVGGVFTSVGK 440
NSW2011  331 GTDGPCKIPISSVASLNDLTPVGRLVTVNPFVSVSTANAKVLIELEPPFGDSYIVVGRGEQQINHHWHKSGSSIGKAFTATLKGAQRLAALGDTAWDFGSVGGVFTSVGK 440
GU0631   331 GTDG   332   ASLNDLTPVGRLVTVNPFVSVSTANAKVLIELEPPFGDSYIVVGRGEQQINHHWHKSGSSIGKAFTATLKGAQRLAALGDTAWDFGSVGGVFTSVGK 440
Boort    331 GTDG [illegible] ASLNDLTPVGRLVTVNPFVSVSTANAKVLIELEPPFGDSYIVVGRGEQQINHHWHKSGSSIGKAFTATLKGAQRLAALGDTAWDFGSVGGVFTSVGK 440
GU1009   331 GTDGPCKIPISSVASLNDLTPVGRLVTVNPFVSVSTANAKVLIELEPPFGDSYIVVGRGEQQINHHWHKSGSSIGKAFTATLKGAQRLAALGDTAWDFGSVGGVFTSVGK 440
K6453    331 GTDGPCKIPISSVASLNDLTPVGRLVTVNPFVSVSTANAKVLIELEPPFGDSYIVVGRGEQQINHHWHKSGSSIGKAFTATLKGAQRLAALGDTAWDFGSVGGVFTSVGK 440
P9974    331 GTDGPCKIPISSVASLNDLTPVGRLVTVNPFVSVSTANAKVLIELEPPFGDSYIVVGRGEQQINHHWHKSGSSIGKAFTATLKGAQRLAALGDTAWDFGSVGGVFTSVGK 440
K74015   331 GTDGPCKIPISSVASLNDLTPVGRLVTVNPFVSVSTANAKVLIELEPPFGDSYIVVGRGEQQINHHWHKSGSSIGKAFTATLKGAQRLAALGDTAWDFGSVGGVFTSVGK 440
K68967   331 GTDGPCKIPISSVASLNDLTPVGRLVTVNPFVSVSTANAKVLIELEPPFGDSYIVVGRGEQQINHHWHKSGSSIGKAFTATLKGAQRLAALGDTAWDFGSVGGVFTSVGK 440

NY99     441 AVHQVFGGAFRSLFGGMSWITQGLLGALLLWMGINARDRSIALTFLAVGGVLLFLSVNVHA 501
SP-B     441 AVHQVFGGAFRSLFGGMSWITQGLLGALLLWMGINARDRSIALTFLAVGGVLLFLSVNVHA 501
LP-F     441 AVHQVFGGAFRSLFGGMSWITQGLLGALLLWMGINARDRSIALTFLAVGGVLLFLSVNVHA 501
NSW2012  441 AVHQVFGGAFRSLFGGMSWITQGLLGALLLWMGINARDRSIAFTFLAVGGVLLFLSVNVHA 501
NSW2011  441 AVHQVFGGAFRSLFGGMSWITQGLLGALLLWMGINARDRSIAFTFLAVGGVLLFLSVNVHA 501
GU0631   441 AVHQVFGGAFRSLFGGMSWITQGLLGALLLWMGINARDRSIAFTFLAVGGVLLFLSVNVHA 501
Boort    441 AVHQVFGGAFRSLFGGMSWITQGLLGALLLWMGINARDRSIAFTFLAVGGVLLFLSVNVHA 501
GU1009   441 AVHQVFGGAFRSLFGGMSWITQGLLGALLLWMGINARDRSIAFTFLAVGGVLLFLSVNVHA 501
K6453    441 AVHQVFGGAFRSLFGGMSWITQGLLGALLLWMGINARDRSIAFTFLAVGGVLLFLSVNVHA 501
P9974    441 AVHQVFGGAFRSLFGGMSWITQGLLGALLLWMGINARDRSIAFTFLAVGGVLLFLSVNVHA 501
K74015   441 AVHQVFGGAFRSLFGGMSWITQGLLGALLLWMGINARDRSIAFTFLAVGGVLLFLSVNVHA 501
K68967   441 AVHQVFGGAFRSLFGGMSWITQGLLGALLLWMGINARDRSIAFTFLAVGGVLLFLSVNVHA 501
```

Figure S3. Comparison of the amino acid sequences of the E protein (501 residues) in the WNV_{KUN} and L1 WNV strains NY99. Positions E^{156} and E^{332} are indicated by black frames. The color of residue indicates the property of the side chain. The highlighted residues show minor residues among the strains. We used a software GENETYX version 13 (Nihon Server, Tokyo, Japan) for the analysis.

Figure S4

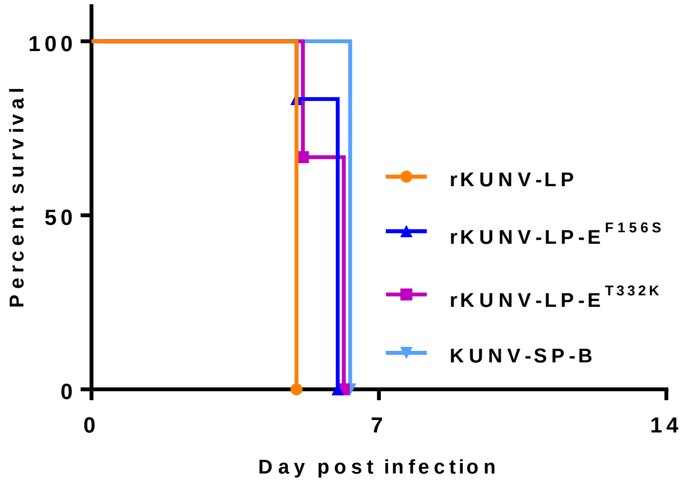

Figure S4. Neurovirulence of the WNV_{KUN} strains. Survival curve of mice inoculated intracerebrally with 1.5×10^4 PFU of the viruses per mouse (n = 6).

Figure S5

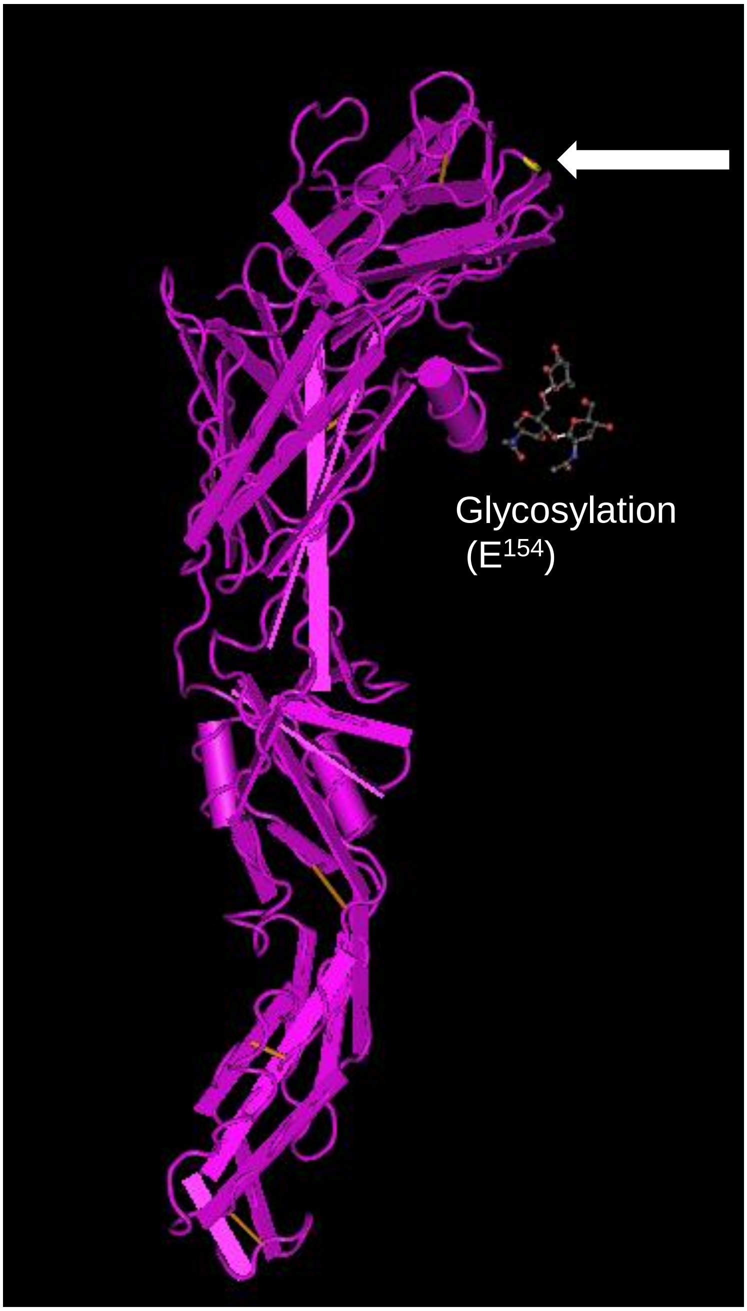

Figure S5. Structure of West Nile virus E protein (PDB ID: 2I69). E^{T332} is indicated by white arrow. The molecular graphic was constructed using Cn3D 4.3 (NCBI).

Table S1. List of oligonucleotides used in this study

Name	Sequence (5’ – 3’)	Applications
KUNV_001f	AGT AGT TCG CCT GTG TGA GCT	PCR amplification
KUNV_2832f	CAT TCT GTT CGC ACC AGA ACT	PCR amplification
KUNV_5721f	TGA GAT AGC CCT CTG TCT GCA	PCR amplification
KUNV_8595f	GAA CTA CCA CGG TAG CTA CGA	PCR amplification
KUNV_3072r	CCA GTA GGA TAG ATC GCT ATG	PCR amplification
KUNV_6013r	GTG ATG GAT TCC TAC CAA TGC	PCR amplification
KUNV_8913r	CAG AGC TGC ATT ACT ATT GAC	PCR amplification
KUNV_11020r	AGA TCC TGT GTT CTC GCA CCA	PCR amplification
IF_KV_OR393_001f	CGG TAC CCG GGG ATC AGT AGT TCG CCT GTG TGA GCT	PCR amplification, Cloning
IF_KV_OR393_2832f	CGG TAC CCG GGG ATC CAT TCT GTT CGC ACC AGA ACT	PCR amplification, Cloning
IF_KV_OR393_5721f	CGG TAC CCG GGG ATC TGA GAT AGC CCT CTG TCT GCA	PCR amplification, Cloning
IF_KV_OR393_8595f	CGG TAC CCG GGG ATC GAA CTA CCA CGG TAG CTA CGA	PCR amplification, Cloning
IF_KV_OR393_3072r	ATG CCT GCA GGT CGA CCA GTA GGA TAG ATC GCT ATG	PCR amplification, Cloning
IF_KV_OR393_6013r	ATG CCT GCA GGT CGA GTG ATG GAC TCC TAC CAA TGC	PCR amplification, Cloning
IF_KV_OR393_8913r	ATG CCT GCA GGT CGA CAG AGC TGC ATT ACT ATT GAC	PCR amplification, Cloning
IF_KV_OR393_11020r	ATG CCT GCA GGT CGA AGA TCC TGT GTT CTC GCA CCA	PCR amplification, Cloning
T7-KUNV_001f	TAA TAC GAC TCA CTA TAG AGT AGT TCG CCT GTG TGA GCT	PCR amplification
KUNV_1429(1433C)f	TAC TCC ACG CAA ACC GGA GCT	Site-directed mutagenesis
KUNV_1428r	GTT CCC ATG CGA TTC CAC GGT	Site-directed mutagenesis
KUNV_1957(1961A)f	GGC AAG GAT GGA CCC TGT AAG	Site-directed mutagenesis
KUNV_1956r	TGT GTA CTG CAG TTC CAA TAC	Site-directed mutagenesis
KUNV_6013r.v2	GTG ATG GAC TCC TAC CAA TGC	Site-directed mutagenesis
KUNV_1279(1285T)f	TGC GGA TTT TTT GGT AAA GGA	Site-directed mutagenesis
KUNV_1293(1285A)r	ACC AAA AAA TCC GCA CCC ATT	Site-directed mutagenesis
KUNV_1906(1913T)f	ACC CCC GTA GAC ACG GGC CAT	Site-directed mutagenesis
KUNV_1920(1913A)r	CGT GTC TAC GGG GGT CCC AAG	Site-directed mutagenesis
KUNV_2278(2285G)f	GTG GGA AGG GCT GTC CAT CAA	Site-directed mutagenesis
KUNV_2292(2285C)r	GAC AGC CCT TCC CAC GGA GGT	Site-directed mutagenesis
WNVcom.3451f	GGH TGT TGG TAT GGH ATG GA	Real time RT-PCR
WNVcom.3590r	TC CTG GGT GGC CAA GAA CAC	Real time RT-PCR
WNV_3538p	FAM-ATG ATT GAY CCT TTT CAG YTG GGC CTT CTG-TAMRA	Real time RT-PCR (probe)

Table S2. P values in Figure 4

Cell (panel)	Virus strain compared	P value			
		Day 1	Day 2	Day 3	Day 4
Vero (C)	rKUN-LP vs. rKUNV-LP-E^{F156S}	0.0019	<0.0001	0.0310	Not tested
	rKUNV-LP vs. rKUNV-LP-E^{T332K}	0.0187	<0.0001	0.0028	Not tested
C6/36 (D)	rKUN-LP vs. rKUNV-LP-E^{F156S}	0.0067	> 0.05	<0.0001	0.0001
	rKUNV-LP vs. rKUNV-LP-E^{T332K}	0.0054	0.0033	<0.0001	<0.0001
IMR-32 (E)	rKUN-LP vs. rKUNV-LP-E^{F156S}	> 0.05	> 0.05	0.0010	<0.0001
	rKUNV-LP vs. rKUNV-LP-E^{T332K}	<0.0001	0.0010	0.0001	<0.0001
Neuro-2a (F)	rKUN-LP vs. rKUNV-LP-E^{F156S}	0.0118	> 0.05	0.0023	0.0091
	rKUNV-LP vs. rKUNV-LP-E^{T332K}	0.0208	0.0113	0.0050	0.0038
